# Novel application of microdissection tungsten needle in total thyroidectomy with central neck dissection for papillary thyroid carcinoma

**DOI:** 10.3389/fsurg.2022.896275

**Published:** 2022-08-26

**Authors:** Weijie Zheng, Shan Zhu, Yimin Zhang, Zhong Wang, Shichong Liao, Shengrong Sun

**Affiliations:** Department of Breast and Thyroid Surgery, Renmin Hospital of Wuhan University, Wuhan, China

**Keywords:** microdissection tungsten needle, harmonic scalpel, energy-based devices, total thyroidectomy, central neck dissection, papillary thyroid carcinoma, hemostasis

## Abstract

**Background:**

Energy-based devices (EBD) have been popularized in thyroidectomy worldwide. Microdissection tungsten needle (MDTN) is characterized by the ultra-sharp tip providing safe and meticulous dissection with effective hemostasis. However, little study has applied MDTN in thyroidectomy.

**Methods:**

This retrospective study compared clinical data of the patients who underwent total thyroidectomy (TT) with central neck dissection (CND) using MDTN, harmonic scalpel (HS), and conventional electrocautery (CE). We assessed outcomes related to surgical efficacy and safety. The injury degree of tissue was assessed by biochemical indicators and early-stage inflammatory factors in the drainage fluid. Histological sections of the thyroid specimens were evaluated to compare levels of thermal damage by the three EBD.

**Results:**

There was a significant decrease in the intraoperative blood loss, operation time and 24-hour drainage volume in the MDTN group compared to the CE group. The total drainage volume, duration of drainage, and average length of stay of the MDTN group were less compared to the CE group though they did not reach statistical significance. No disparity was observed between the MDTN group and HS group in these variables. Total costs were not significantly different among these groups. The incidence of recurrent laryngeal nerve (RLN) injury was the lowest using MDTN compared to the CE (*P* = 0.034) and HS (not significant). No statistical differences were observed among these groups regarding postoperative wound pain and infection, hypoparathyroidism, and postoperative hemorrhage. Analysis of biochemical indicators showed a lower level of hemoglobin in the MDTN and HS group than the CE group (*P* = 0.046 and 0.038, respectively) and less triglyceride in the HS group than the MDTN and CE group (*P* = 0.002 and 0.029, respectively) but no significant difference in cholesterol level in these groups. Early-stage inflammatory factors including TNF-α and IL-6 showed significantly higher concentration in the CE group than the MDTN and HS group. Histological sections of thyroid specimens revealed that MDTN caused the lowest degree of thermal damage followed by HS then CE.

**Conclusion:**

MDTN exhibited comparable surgical efficacy and safety outcomes as HS in thyroidectomy. Therefore, MDTN is a safe and viable alternative for hemostasis in thyroidectomy.

## Introduction

Thyroid carcinoma is the most common head and neck tumor with an increasing global incidence in recent years (1), and papillary thyroid carcinoma (PTC) remains the most common histological subtype of the new diagnoses (2). Total thyroidectomy (TT) is the time-tested primary treatment modality of PTC (3).

Thyroidectomy was originated in the 19th century. As the thyroid gland is richly vascularized and adjacent to several important anatomical structures, thyroidectomy was a particularly risky surgery with a complication rate up to 50% and a mortality rate up to 20% in that period (4). It was not until the late 20th century that the surgical complication rate decreased to merely 1% with the popularization of suture and ligation techniques and then the invention of energy-based devices (EBD) for hemostasis. Additionally, with the accumulation of surgical experience, surgeons with high volume can further reduce the complication rate (2, 5–7).

Though the traditional suture and ligation method helps to achieve the purpose of careful hemostasis, it is time-consuming and excessive ligation to the adjacent tissue may cause incidental injury to the nerves and parathyroid glands. Conventional electrocautery (CE) has a relatively large and blunt tip which may increase the operating power output, causing more severe tissue damage and more diffusion of lateral heat (8). Currently, the harmonic scalpel (HS) has been applied for hemostasis and dissection in thyroid surgery as its excellent surgical efficacy has been well-documented (9–16), but it increases the medical costs to a certain extent in our country. The major mechanism of HS is breaking the tertiary structure hydrogen bonds of protein in tissue by emitting mechanical vibration with a frequency of 55.5 kHz (17). Although the majority of researchers have been prone to recommend HS for hemostasis in thyroid surgery, there is no solid evidence to decide which is the best hemostatic modality overall, while certain research aroused concerns about the safety of utilizing HS in thyroid surgery (10, 18, 19). Therefore, more investigation and further development of EBD are still warranted.

Microdissection tungsten needle (MDTN) is a surgical device featured with its ultra-sharp needle using tungsten alloy with a tip radius of curvature less than 10 microns. Based on the mechanism of high-frequency electrocautery, it combines the advantages of scalpel and electrosurgery with lower wattage input and more concentrated thermal delivery to reduce thermal injury compared with CE. It provides meticulous dissection comparable to scalpel while reducing the risk of surgical complication development, contributing to the postoperative recovery of patients. Furthermore, it is more cost-efficient compared to HS, thus it has a wide application prospect in surgery. MDTN has been applied in several fields of surgery with good performance, especially orthopedic and otorhinolaryngologic surgery (20–26). However, little research has been conducted on the application of MDTN in thyroid surgery. This research aimed to compare the surgical efficacy and safety of MDTN with HS and CE for the first time in patients who underwent TT with central neck dissection (CND) for PTC. Additionally, the traumatic degree of soft tissue caused by these devices was investigated. We present the following article in accordance with the STROBE reporting checklist.

## Materials and methods

### Study design

A retrospective study was conducted on clinical records of patients who were diagnosed with PTC and underwent TT with bilateral CND in our department from March 2018 to June 2019. The exclusion criteria included: (I) patients undergoing unilateral thyroidectomy; (II) patients without bilateral CND operation; (III) history of chronic diseases of vital organs; (IV) history of hematological diseases; (V) patients undergoing lateral neck dissection; (VI) history of preoperative hyperthyroidism; (VII) history of anticoagulant agent use; (VIII) history of thyroid surgery; (IX) patients with follow-up less than 6 months or withdraw. Patients involved were divided into MDTN (BT-500V, BROVET®, China), HS (GEN300, Johnson & Johnson), and CE (SM-ZXDJ-A61, SM company, China) groups according to the use of EBD modality in surgery. The electrosurgical generator of MDTN and CE was Force FX of COVIDEN company and the generator of HS was Ethicon Endo-Surgery from Johnson & Johnson company. MDTN and CE were generally applied by the CUT mode and the output power were 15 and 30 w, respectively. The study was conducted in accordance with the Declaration of Helsinki (as revised in 2013). All patients signed informed consent. The study protocol was approved by the Ethics Committee of Renmin Hospital of Wuhan University (item ID: ADRY2021-K032).

### Surgical procedures

All the patients included in the study underwent TT with bilateral CND under the standard surgical procedure similar to the description by Ramouz et al. (27). CND was performed after TT. During surgery, MDTN, HS, or CE was utilized as the major modality for hemostasis and dissection. Note that if the hemostatic effect was insufficient, ligation and suture were applied. A drainage tube was routinely placed before closure. The dissected specimens of the thyroid were routinely sent for pathological diagnosis after surgery. The H&E staining slices of the thyroid specimens were collected to determine the degree of tissue damage by the three EBD. Analysis of histological changes of the thyroid margin was conducted by a pathologist who was blinded to the EBD usage corresponding to the specimens.

### Clinical data

Demographic characteristics including age, sex, BMI (body mass index), history of hypertension, and diabetes mellitus were recorded accordingly. The number of intraoperative dissected lymph nodes and metastatic lymph nodes (histologically confirmed after surgery) was also recorded.

The surgical efficacy indicators included: intraoperative blood loss volume, operation time, 24-hour drainage and total drainage volume, duration of postoperative drainage, average length of hospital stay, and total medical costs. Intraoperative blood loss was defined as the volume of fluid in the negative pressure suction device. The operation time was defined as the time between skin incision and complete closure. The indication of drainage tube removal was 24-hour drainage volume less than 30 ml. Drainage volume was recorded 24 h after surgery and by the time of drainage tube removal.

The safety assessment consisted of two parts: general complications including surgical wound pain and infection, and complications specific to thyroid surgery including recurrent laryngeal nerve (RLN) injury, postoperative hypoparathyroidism, and postoperative hemorrhage. Surgical wound pain level was assessed 24 h after surgery by visual analog scale (VAS) from 0 to 10 (0 for no pain; 1–3 for mild pain; 4–6 for moderate pain; 7–10 for severe pain). For each group, the number of patients reporting moderate or severe pain was documented accordingly. Surgical wound infection was defined as the local symptoms including erythema, swelling, warmth, pain, and purulent secretion in the wound. RLN injury was defined as postoperative hoarseness or vocal cord paralysis revealed by laryngoscopy. Postoperative hypoparathyroidism was defined as either PTH level lower than the normal range (18.5–88 pg/ml in our hospital) or hypocalcemia after surgery along with the symptom of numbness or cramping in hands and feet. Postoperative hemorrhage was defined as surgical wound swelling with symptoms of tracheal compression such as suffocation.

Since March 2019, the drainage fluid of 37 consecutive patients involved (13 from MDTN group, 16 from HS group, 8 from CE group) were collected by the day of drainage tube removal and sent for laboratory examination, including biochemical tests (hemoglobin, cholesterol, and triglyceride) and early-stage inflammatory factors (TNF-α and IL-6).

### Statistical analysis

Statistical analysis was performed by SPSS 22.0 software (SPSS Inc., Chicago, IL, USA). Pearson chi-square test or Fisher’s exact test were conducted for categorical variables. Continuous variables were checked for distribution type using histogram and Shapiroe-Wilk test. Continuous variables conformed to the normal distribution were compared by the one-way ANOVA test and expressed as mean ± SD, while those did not conform to the normal distribution were compared by the Kruskal-Wallis ANOVA test and expressed as the median and first-third quartile. The post-hoc pairwise comparison was conducted when the difference was significant. Post-hoc pairwise comparisons were adjusted using a Bonferroni correction. *P* ＜ 0.05 was considered statistically significant. Note that in this paper, P represented the comparison of the three groups, and *P*1, *P*2, *P*3 respectively represented the results of statistical comparison of MDTN group versus HS group, HS group versus CE group, MDTN group versus CE group.

## Results

### Baseline characteristics

A total of 242 patients were involved in this study, including 85 in the MDTN group, 115 in the HS group, and 42 in the CE group. The baseline characteristics showed no significant differences between the three groups (Table 1). Therefore, the three groups are considered comparable.

**Table 1:** Baseline characteristics of the patients.

Variable	MDTN	HS	CE	*P*
Age (years), mean ± SD	46.69 ± 13.13	49.35 ± 12.42	46.90 ± 10.61	0.268
Sex (male/female)	14/71	28/87	9/33	0.401
BMI (kg/m^2^), mean ± SD	23.51 ± 3.27	23.82 ± 2.87	24.56 ± 4.85	0.270
Hypertension (yes/no)	16/69	17/98	3/39	0.220
Diabetes mellitus (yes/no)	10/75	12/103	3/39	0.722
DLN, median (first-third quartile)	5 (4–7)	5 (4–7)	5 (4–7)	0.740
MLN, median (first-third quartile)	0 (0–1)	0 (0–2)	1 (0–2)	0.080

P, comparison of the three groups; MDTN, microdissection tungsten needle; HS, harmonic scalpel; CE, conventional electrocautery. SD, standard deviation; BMI, body mass index; DLN, dissected lymph nodes; MLN, metastatic lymph nodes.

### Surgical efficacy assessment

The comparison of surgical effects of the three groups was demonstrated in Table 2. The intraoperative blood loss volume of the MDTN and HS group was significantly less than the CE group (*P*2 < 0.001, *P*3 = 0.002). The operation time of the MDTN and HS group was significantly shorter than the CE group (*P*2 = 0.061, *P*3 = 0.029). Consistently, compared to the CE group, MDTN and HS group had less 24-hour drainage volume (*P*2 < 0.001, *P*3 = 0.020), less total drainage volume (*P*2 = 0.003, *P*3 not significant), shorter duration of postoperative drainage (*P*2 = 0.017, *P*3 not significant), and shorter average length of hospital stay (*P*2 = 0.017, *P*3 not significant). There was no significant difference in the indicators above between the MDTN group and the HS group. The total medical costs of the three groups were not statistically significant (*P* = 0.280).

**Table 2:** Surgical efficacy comparison of the three groups.

Variable	MDTN	HS	CE	*P*	*P*1	*P*2	*P*3
Blood loss (ml)	45 (40–56)	46 (37–56)	56.5 (44.75–67)	<0.001***	1.000	<0.001***	0.002**
Operation time (minutes)	78 (73–87)	79 (72–85)	85 (77.75–92.5)	0.014*	1.000	0.016*	0.029*
24-hour drainage (ml)	65 (54.5–75)	60 (50–70)	76 (58.75–88)	<0.001***	0.502	<0.001***	0.020*
Total drainage (ml)	100 (89–125.5)	90 (80–110)	120 (89.75–154.25)	0.005**	0.428	0.003**	0.139
Duration of drainage (days)	2 (2–3)	2 (2–3)	3 (2–3)	0.019*	1.000	0.017*	0.067
Average length of stay (days)	4 (4–5)	4 (4–5)	5 (4–5)	0.019*	1.000	0.017*	0.067
Total costs (×10^4^ CNY)	2.30 (2.23–2.67)	2.32 (2.22–2.56)	2.38 (2.26–2.55)	0.280	NS	NS	NS

*P*: comparison of the three groups; *P*1: MDTN vs. HS; *P*2: HS vs. CE; *P*3: MDTN vs CE. MDTN, microdissection tungsten needle; HS, harmonic scalpel; CE, conventional electrocautery. All the data in Table 2 were presented as median (first-third quartile). *P*1, *P*2 and *P*3 were adjusted using a Bonferroni correction. *P < 0.05; **P < 0.01; ***P < 0.001; NS, not significant.

### Safety assessment

The comparison of safety assessments was demonstrated in Table 3. No case of RLN injury occurred in the MDTN group, which was significantly less than that of the CE group (*P*3 = 0.034). While the incidences of RLN injury were 0.87% and 7.14% in the HS group and CE group, respectively. No significant difference was shown regarding other complications including surgical wound pain, wound infection, hypoparathyroidism, and postoperative hemorrhage. Both MDTN group and CE group had one case of postoperative hemorrhage, of which the case of MDTN group resulted from an accidental tumble during off-bed activity the day after surgery and was resolved by strengthened local compression, while the case of CE group developed symptoms of airway compression on the day after surgery and underwent an emergency operation for hematoma evacuation. There was no case of lymphatic leakage in all the patients.

**Table 3:** Surgical complications comparison of the three groups.

Variable	MDTN	HS	CE	*P*	*P*1	*P*2	*P*3
Surgical wound pain	64 (75.29)	89 (77.39)	33 (78.57)	0.903	NS	NS	NS
Surgical wound infection	2 (2.35)	2 (1.74)	1 (2.38)	1.000	NS	NS	NS
RLN injury	0 (0)	1 (0.87)	3 (7.14)	0.017*	1.000	0.059	0.034*
Hypoparathyroidism	0 (0)	4 (3.48)	2 (4.76)	0.104	NS	NS	NS
Postoperative hemorrhage	1 (1.19)	0 (0)	1 (2.38)	0.274	NS	NS	NS

*P*: comparison of the three groups; *P*1: MDTN vs. HS; *P*2: HS vs. CE; *P*3: MDTN vs. CE. MDTN, microdissection tungsten needle; HS, harmonic scalpel; CE, conventional electrocautery. RLN, recurrent laryngeal nerve. All the data in table 3 were presented as *n* (%). *P*1, *P*2 and *P*3 were adjusted using a Bonferroni correction. *P < 0.05; NS, not significant.

### Laboratory examination of drainage fluid

**Figure 1:**
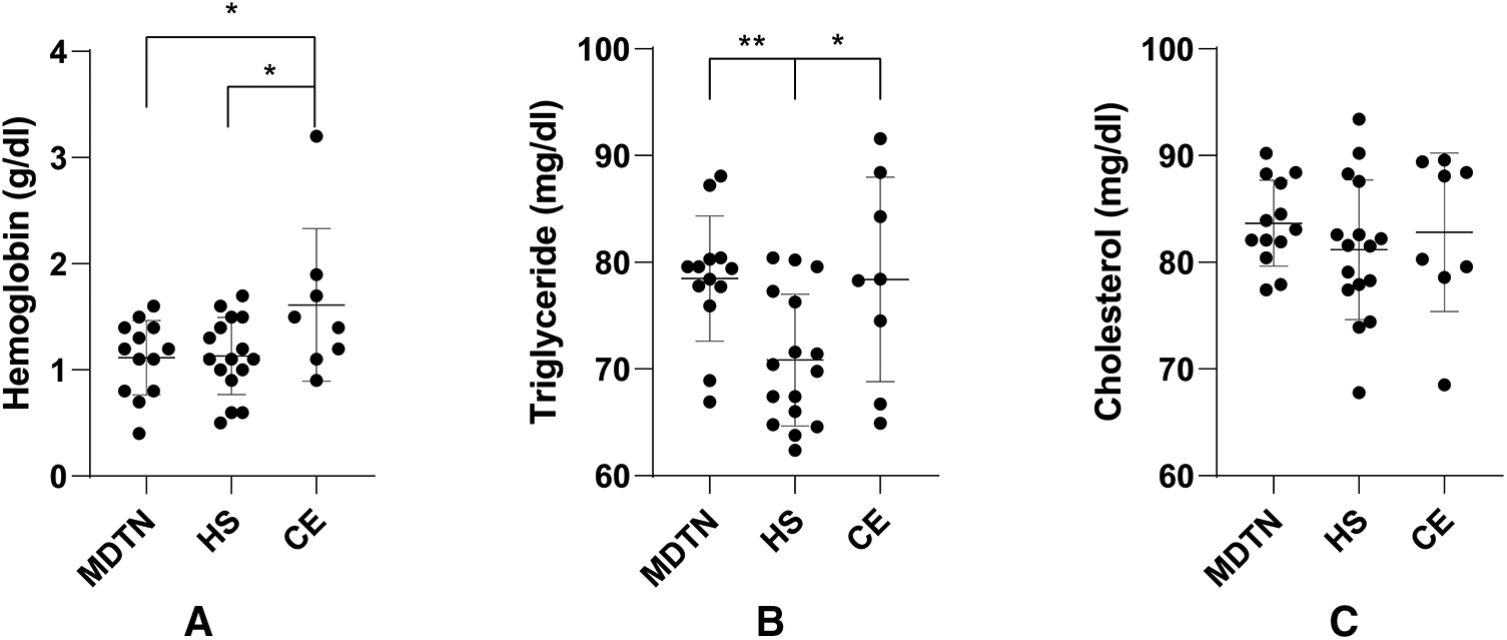
Comparison of biochemical indicators in the drainage fluid from the three groups. (**A**) Hemoglobin concentrations in the drainage fluid; (**B**) Triglyceride concentrations in the drainage fluid; (**C**) Cholesterol concentrations in the drainage fluid. *P < 0.05; **P < 0.01.

**Figure 2:**
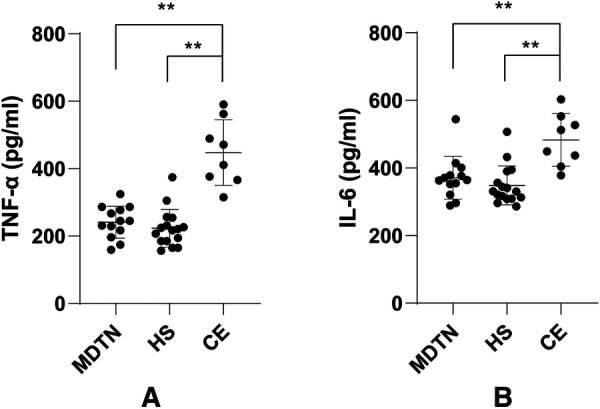
Comparison of early-stage inflammatory factors in the drainage fluid from the three groups. (**A**) TNF-α levels in the drainage fluid; (**B**) IL-6 levels in the drainage fluid. **P < 0.01.

Among all the patients involved, 37 cases (13 from the MDTN group, 16 from the HS group, 8 from the CE group) were analyzed with biochemical tests (Figure 1) and early-stage inflammatory factors (Figure 2) of the drainage fluid. It was indicated that hemoglobin was significantly lower in the MDTN and HS group compared to the CE group (*P*2 = 0.046, *P*3 = 0.038), and the content of triglyceride was significantly lower in the HS group compared to the MDTN and CE group (*P*1 = 0.002, *P*2 = 0.029). There was no significant difference among the three groups regarding the level of cholesterol. In terms of the early-stage inflammatory factors, levels of TNF-α and IL-6 in the drainage fluid were significantly higher in the CE group compared to the other two groups (*P* < 0.001).

### Histological section of thyroid specimens

**Figure 3:**
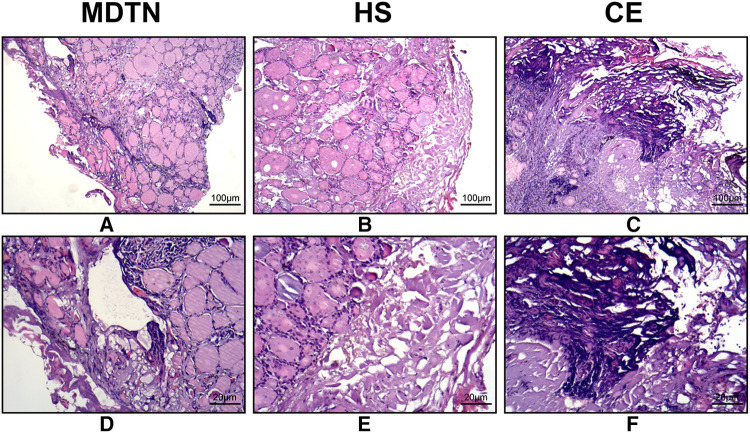
Histological sections (H&E staining) of the surgical margins of thyroid specimens from the three groups. The scale bars were included in the figures. (**A,B**) MDTN group; (**C,D**) HS group; (**E,F**) CE group.

Figure 3 illustrated the H&E staining results of surgical margins of thyroid specimens from the three groups. It was observed that the thyroid margins in MDTN and HS group had a lower degree of thermal damage compared with the CE group. MDTN group (Figures 3A,B) exhibited the least degree of thermal damage because most of the glandular follicles remained structural integrity with continuous follicular epithelium surrounding the colloid, and the width of tissue necrosis was relatively narrower. In the HS group (Figures 3C,D) the follicular structures showed a higher level of damage by coagulative necrosis where more follicles were distorted and the colloid inside showed solid concentration with deeper staining color compared to the MDTN group, but parts of the structure remained relatively complete. In the CE group (Figures 3E,F) thyroid margin was seriously damaged as a wide range of nuclear pyknosis, fragmentation, and karyolysis were observed and tissue carbonization was the severest in all groups, with little intact follicular structure remaining.

## Discussion

In recent decades, a series of EBD have been invented and employed in thyroid surgery to provide a less invasive manner of dissection and hemostasis (17). However, it remains controversial whether EBD performs better than traditional hemostatic methods in all aspects and how to choose among various EBD according to their profiles of efficacy and safety, which prompts the need for continued investigation of EBD.

**Figure 4:**
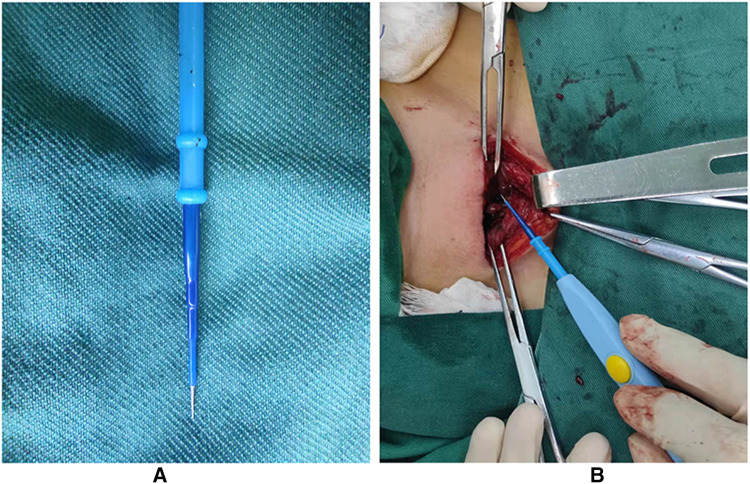
The application of MDTN in thyroidectomy. (**A**) The sharp tungsten tip of MDTN; (**B**) The operative view of thyroidectomy utilizing MDTN.

MDTN is an energy-based device characterized by the ultra-sharp tip that combines the advantages of scalpel and electrocautery while providing effective hemostasis (Figure 4A). However, little research has utilized MDTN in thyroid surgery. Therefore, we conducted this research on the novel application of MDTN in patients who underwent TT with CND with histological diagnosis of PTC (Figure 4B). We assessed comprehensive outcomes including three major contents, which were surgical efficacy, safety outcomes, and trauma degree comparison. Overall, it was discovered that MDTN was comparable to HS, and both devices performed better than CE.

Concerning the surgical efficacy, our study found that MDTN and HS exhibited better profiles in intraoperative blood loss volume, operation time, 24-hour drainage and total drainage volume, days of postoperative drainage, average stay in hospital compared to CE. The analysis of total drainage volume, days of postoperative drainage and average stay in hospital showed that the difference between MDTN group and CE group did not reach statistical significance after adjusting the P value by Bonferroni correction, while the P values were statistically significant before correction. Bonferroni correction is one of the most commonly used methods for multiple hypothesis testing with the major purpose of controlling the probability of type I error. However, many researchers pointed that Bonferroni correction could be relatively conservative and may increase the type II error rate (28). Therefore, it is inappropriate that any scientific inference relies merely on the P value or the statistical significance without consideration of the clinical evidence provided by the data (28). In this case of research, there was an obvious decrease in these surgical variables by using MDTN comparing CE, and the data between HS group and MDTN group were relatively close comparing CE group. Therefore, it is reasonable to conclude that MDTN improved the surgical efficacy to a certain extent compared to CE, but research with larger samples is needed to verify this conclusion. Several prospective research (12–15) and meta-analyses (9, 11) concluded that HS had equally good or superior surgery outcomes compared to other hemostatic techniques and devices. Therefore, HS has been popularized in thyroid surgery worldwide. In this study, we observed a decrease in intraoperative blood loss volume and operation time by utilizing MDTN or HS compared to CE, which proved that MDTN not only achieved better hemostatic effect but can reduce the time for hemostasis and improve the surgical efficiency. In our opinion, it was majorly attributed to the meticulous dissection provided by MDTN when separating the thyroid capsule. Besides, MDTN and HS similarly reduced the volume and duration of postoperative drainage, indicating that MDTN can lessen the damage to soft tissue and lymphatic system in the operation area, thus facilitating the recovery progress and shortening the length of stay of patients. Some research pointed out that the application of EBD increased the costs of treatment (29, 30). Herein we found that there was no significant difference among three groups regarding total in-hospital costs. Further economic efficiency analysis is needed to compare these EBD.

In the safety assessment part, we stated that MDTN did not increase the risk of postoperative complications compared to HS and CE, and even exhibited a better profile in RLN protection. There was no case of RLN injury in the MDTN group while the rates were 0.87% and 7.14% in the HS group and CE group, respectively, but all recovered within three months after surgery. Since EBD can cause injury to RLN due to their thermal effect, numerous researches have been dedicated to exploring the thermophysical properties and safety capability of various EBD to establish standardized guidance for scrupulous and safe utilization of EBD (31). Most of the related studies showed that HS can achieve a good hemostatic effect without increasing the incidence of injury to adjacent nerves (9, 11, 15). One preclinical study stated that HS was safer than CE as it caused less damage to thyroid tissue and RLN in a canine model (8). Our findings showed that MDTN may cause less thermal injury to RLN with no case of RLN paralysis reported. It was probably attributed to the sharp needle tip of MDTN that required less energy and wattage input to accomplish fine dissection, which may reduce lateral thermal energy diffused to RLN and other structures. However, this hypothesis should be testified by a series of preclinical studies and prospective clinical trials.

The main causes of parathyroid gland injury are incidental removal of the gland and damage to the blood supply. In this study, there was no case of postoperative hypoparathyroidism in the MDTN group while four were in the HS group (3.48%) and two were in the CE group (4.76%), of which one patient in the HS group did not recover by six-month follow-up after surgery. The decreased rate of hypoparathyroidism by using MDTN may be attributed to some reasons. First, the sharp and thin tip of MDTN enabled the operator to dissect the thyroid gland while circumventing the parathyroids and their vascular supply. Second, MDTN may reduce lateral thermal damage to the parathyroid gland because the sharp tip of MDTN delivered energy in a more concentrated manner during cauterization. Although these differences failed to attain statistical significance (*P*1 = 0.138, *P*3 = 0.108), such disparity may be more obvious with a larger volume of participants. According to the previous research, the rate of postoperative transient hypoparathyroidism was reported about 19%–38% (32). The discrepancy between the results in our study and the previous research could be ascribed to the following clinical factors. First, all the patients who underwent total thyroidectomy in our department are routinely prescribed the oral calcium supplements after surgery, which effectively reduced the incidence of transient postoperative hypocalcemia and the related symptoms. Second, in our department, a series of techniques such as carbon nanoparticle suspension negative imaging (19), indocyanine green (ICG) fluorescence imaging (33), and parathyroid hormone test strip (34) are utilized for the identification and protection of the parathyroid gland. Based on this experience in our department, a decrease in postoperative hypoparathyroidism rate was observed in the patients.

According to previous research, the application of EBD was associated with decreased risk of developing postoperative neck hematoma (35, 36). As we know, neck hematoma is a dangerous but rare surgical complication in thyroid surgery, which is difficult to be evaluated in a small-volume prospective or retrospective study. Herein, only one patient in the CE group developed neck hematoma and received emergency hemostasis, while the patient in the MDTN group who developed postoperative hemorrhage resulted from an accidental tumble and was resolved by local compression. Therefore, the discrepancy in the risk of neck hematoma between the three groups did not reach statistical significance and a larger investigation was still warranted.

The general complications including postoperative pain and wound infection showed no statistical disparity in these groups. Among all the patients included, 75.29% of the MDTN group, 77.39% of the HS group and 78.57% of the CE group reported a moderate or higher level of pain and were prescribed intravenous administration of analgesics. Accordingly, 2.35%, 1.74%, 2.38% of the three groups above had wound infection but all recovered after prescription of antibiotics.

In terms of laboratory examination of 37 samples of drainage fluid, we found that hemoglobin levels in MDTN and HS group significantly decreased compared to CE group, which was in accordance with the result of blood loss volume, indicating that MDTN could achieve comparable hemostatic efficiency as HS to reduce postoperative exudation. It was noticed that triglyceride level was significantly less in the HS group than that in MDTN and CE group. We conjectured that such phenomenon may provide a limited clue that HS performed better regarding lymphoid tissue closure during neck dissection since triglyceride is one of the major components of lymph. However, given the limited volume of our examination, such a hypothesis requires a larger test to elucidate the physiological basis of drainage reduction with HS application.

To further compare the damage degree of tissue caused by these EBD, we tested the drainage fluids of 37 patients for TNF-α and IL-6 levels. It is well-known that TNF-α and IL-6 are classic proinflammatory cytokines functioning in many biological processes such as inflammation, infection, and wound healing (37). Surgical trauma stimulates macrophages and monocytes to activate and secrete TNF-α and IL-6 in the operated tissue, which can induce the cellular production of elastase and matrix metalloproteinase contributing to wound healing. Therefore, we supposed that examination of the drainage fluid from the surgical wound can potentially evaluate the tissue trauma (38). In this study, TNF-α and IL-6 levels were markedly lower in MDTN and HS group than in the CE group, indicating at the cellular level that MDTN and HS reduced the traumatic degree of tissue. However, considering the small number of samples included (37 samples in total), a larger sample size for investigation is warranted to confirm these findings.

Additionally, we performed H&E staining with several specimens of thyroid gland to gain an insight into the thermal damage to soft tissue caused by these EBD at a pathological level. Previous studies suggested that a more severe degree of mechanical and thermal destruction of the tissue would lead to the prevention of an appropriate pathological assessment (39, 40). Some researchers have previously proven MDTN caused very little tissue distortion during fine dissection of skin in a rat model (41). These studies are in accordance with our findings that MDTN reduced tissue necrosis and distortion in thyroid follicular structure, followed by HS with relatively more distorted follicle near the margin, while CE led to extensive carbonization and eschar formation. Thus, it can be concluded that MDTN best preserved the pathological integrity for thyroid cancer diagnosis. However, it should be clarified that only in the cases when the tumor invaded the thyroid capsule that different EBD may affect the diagnosis by pathologist.

Overall, MDTN has overcome the disadvantages of CE and illustrated equally good surgical efficacy and safety outcomes as HS in thyroidectomy. Additionally, MDTN provided a more integrated histological section of thyroid specimens for pathologists. Therefore, MDTN is an advantageous and applicable option for surgeons. However, given the fact that the experience of surgeons is in strong association with surgical outcomes (2), it should be highlighted that surgeons ought to choose appropriate hemostatic modalities according to their experience and awareness of device properties, and flexibly combined them when necessary to achieve optimal outcomes.

There were several limitations in our study. First, since it was a single-center retrospective study, the conclusions were limited in their feasibility compared to randomized controlled trials (RTC). Therefore, we have planned to conduct a prospective study in our center to further investigate the feasibility and safety of the MDTN application. Second, the number of patients enrolled was relatively small, and we will enlarge the scale in the next prospective study. Third, we limited the study object to PTC patients with TT with CND. On one hand, it was beneficial to reduce the influence of surgical methods and pathological types on the results. On the other hand, the application of MDTN in thyroid surgery treating other types of diseases such as benign thyroid goiter still needed investigation.

## Conclusion

This is the first clinical study of applying MDTN in thyroidectomy. MDTN can provide meticulous dissection and effective hemostasis while reducing adverse thermal injury to soft tissue. In this study, MDTN exhibited comparable surgical efficacy and safety as HS. Therefore, MDTN is a novel and feasible hemostatic modality for thyroid surgery.

## Data Availability

The original contributions presented in the study are included in the article/Supplementary Material, further inquiries can be directed to the corresponding author/s.
